# DreamTel; Diabetes risk evaluation and management tele-monitoring study protocol

**DOI:** 10.1186/1472-6823-9-13

**Published:** 2009-05-09

**Authors:** Sheldon W Tobe, Joan Wentworth, Laurie Ironstand, Susan Hartman, Jackie Hoppe, Judi Whiting, Janice Kennedy, Colin McAllister, Alex Kiss, Nancy Perkins, Lloyd Vincent, George Pylypchuk, Richard Z Lewanczuk

**Affiliations:** 1 Division of Nephrology, Sunnybrook Health Sciences Centre, University of Toronto, Toronto, Canada; 2 Battelford Tribal Council Health Services Inc. North Battleford, Saskatchewan, Canada; 3 LiveWell Diabetes Program at Muskeg Lake, Saskatchewan, Canada; 4 Perspect Management Consulting, Regina, Saskatchewan, Canada; 5 Division of Statistical Design and Analysis, Institute of Clinical Evaluative Sciences, Toronto, Canada; 6 Division of Nephrology, St Paul's Hospital, University of Saskatoon, Saskatoon, Canada; 7 Division of Endocrinology and Metabolism, University of Alberta, Alberta, Canada

## Abstract

**Background:**

The rising prevalence of type 2 diabetes underlines the importance of secondary strategies for the prevention of target organ damage. While access to diabetes education centers and diabetes intensification management has been shown to improve blood glucose control, these services are not available to all that require them, particularly in rural and northern areas. The provision of these services through the Home Care team is an advance that can overcome these barriers. Transfer of blood glucose data electronically from the home to the health care provider may improve diabetes management.

**Methods and design:**

The study population will consist of patients with type 2 diabetes with uncontrolled A1c levels living on reserve in the Battlefords region of Saskatchewan, Canada. This pilot study will take place over three phases. In the first phase over three months the impact of the introduction of the Bluetooth enabled glucose monitor will be assessed. In the second phase over three months, the development of guidelines based treatment algorithms for diabetes intensification will be completed. In the third phase lasting 18 months, study subjects will have diabetes intensification according to the algorithms developed.

**Discussion:**

The first phase will determine if the use of the Bluetooth enabled blood glucose devices which can transmit results electronically will lead to changes in A1c levels. It will also determine the feasibility of recruiting subjects to use this technology. The rest of the Diabetes Risk Evaluation and Management Tele-monitoring (DreamTel) study will determine if the delivery of a diabetes intensification management program by the Home Care team supported by the Bluetooth enabled glucose meters leads to improvements in diabetes management.

**Trial Registration:**

Protocol NCT00325624

## Background

### Diabetes and its impact on the community

Diabetes is a systemic disorder resulting in abnormally high blood glucose levels. These elevated levels impact directly and indirectly on every part of the body. Diabetes is the number one cause of end stage renal disease (ESRD), preventable blindness, as well as a major cause of premature heart disease, stroke, limb amputation and preventable hospital admissions[1]. Prevention of type 2 diabetes through primary control programs is society's ultimate goal but current evidence indicates that this goal is failing as rates of diabetes are rising rapidly. For example in the Province of Ontario, prevalence rates of diabetes rose by 69% to 8.8% in the 10 year period ending 2005[2]. Secondary prevention strategies to help reduce the complication rate in this burgeoning population of people with diabetes are desperately needed. Control of blood glucose levels prevents new microvascular disease and has recently been shown to have a legacy effect and is a central focus of diabetes management programs[3], in addition to the significant health benefits of blood pressure and lipid control. The optimal degree of blood glucose normalization has recently come into question. While the ADVANCE study demonstrated a reduction of microvascular but no significant reduction of macrovascular events with normalization of A1c to below 7.0%[4], the ACCORD study, also targeting normalization of A1c below 7.0%, found an elevated mortality and no reduction of cardiovascular events[5].

For those who develop diabetes, control of blood glucose is important to prevent new microvascular target organ damage. Blood pressure control prevents both micro and macrovascular damage and lipid control macrovascular changes. Two examples of health care initiatives to control blood glucose levels in people with diabetes are now the standard of care: The development and wide dissemination of the Canadian Diabetes Association's Clinical Practice Guidelines[1] and the widespread dissemination of Diabetes Education and self-management[6]. The guidelines are evidence based and described in detail and, if followed, would lead to control of blood glucose levels. The guidelines are largely based on outcomes research carried out in carefully controlled settings. The challenge is in implementing them in a real world setting. Diabetes education centers and Diabetes Educators are a necessary part of the team for intensive diabetes management[6], but are still not accessible to many people living in rural and Northern areas. Furthermore there are only a few Diabetes Education Centers designed specifically for First Nations peoples. The closest in 2007 for the people living in Northern Saskatchewan where this study takes place, is more than a 3 hour drive away in Edmonton. However, Home Care teams which provide home visits from nurses and even dietitians and other members of the multidisciplinary team are able to access virtually all communities. Traditionally Home Care teams have provided wound and dressing care. However, the Home Care team can include Diabetes Educators that can deliver evidence based therapy as part of a chronic disease management program for diabetes.

We believe that this study protocol has relevance with respect to the development of a new community-based approach to the control of blood glucose. We have recently demonstrated that the Home Care team (which provides supportive care provided in the patient's home by healthcare professionals) of the Battlefords Tribal Council Indian Health Services, can improve blood pressure control using a chronic disease management model for multiple drug therapy[7]. We believe that a chronic disease management model linking the Home Care Team including Diabetes Educators[6] with their clients and the primary care providers to try to bring A1c to 7.0% can improve blood glucose control safely. Combining elements of chronic disease management including provider education, patient education, clinical practice guidelines, decision support and disease measurement is expected to lead to lasting improvements in diabetes management[8]. Use of the Bluetooth enabled glucose meters may be able to enable diabetes intensification in a safe and effective manner, allowing the Home Care team to review their clients with access to their client's self monitored blood glucose results.

### Diabetes, First Nations and kidney disease

End stage kidney disease (ESRD) comprises an enormous public health burden, with an incidence and prevalence that are increasing alarmingly. For example, between 1998 and 2002, the prevalence of ESRD in Canada increased 33%, while the incidence increased 17%[9]. In Canada, 4.7% of all new dialysis starts are identified as Aboriginal while in the province of Saskatchewan 19.7% of new dialysis starts are identified as aboriginal[10]. In a study of the aboriginal population of Australia, Hoy found an incidence of ESRD in this population ten times greater than the incidence in the general population [11]. First Nations Peoples are at higher risk for ESRD, largely due to diabetes [12-15]. The prevalence of diabetic nephropathy is much higher among Aboriginal Canadians than in the general population, with rates ranging from 25–60%[16,17]. Morrison [18] noted that in the First Nations population of the Sioux Lookout Zone, Northwestern Ontario, that children as young as five years of age have been diagnosed with type 2 diabetes. With diabetes occurring at increasingly younger ages, ESRD is going to become more prevalent in a younger age group with diabetes[16]. Canada is not alone in this respect as the prevalence of kidney disease in the Australian Aboriginal population with diabetes is approaching 50%[19,20]. Hypertension in those with diabetes is a strong predictor of death from diabetic nephropathy [21]. The etiology of the diabetes epidemic in the aboriginal community is multifactorial in nature, including social and societal issues endemic to Western society and those specific to the aboriginal community, as well as genetic factors and are beyond the scope of this project [22].

Unless early and sustained blood glucose control can be achieved, higher rates of diabetes with earlier age of onset will result in more frequent complications from diabetes with resultant higher costs to quality of life and the health care system. Linked to the rising rates of diabetes in Aboriginals are risk factors for and higher rates of cardiovascular disease [23-32]. Kidney disease, now recognized as a significant marker of cardiovascular disease [33-35], may be in part responsible for this and blood glucose control has been demonstrated to be effective in reducing the progression of kidney disease in the setting of diabetes[36]. However, medication alone is insufficient to achieve control. London[37] has demonstrated, in an Australian Aboriginal diabetes study, that health information must also incorporate the contemporary beliefs of the community. Implementation of treatment programs with First Nations peoples must be carried out with sensitivity to cultural mores and beliefs [38-42].

Intensification of Diabetes Management. The current Canadian Diabetes Association Clinical Practice Guidelines recommend similar glycaemic goals for both type 1 and type 2 diabetes and define optimal glycaemic control as an A1c < 7.0% [43]. A stepwise approach is advocated with initial therapy consisting of lifestyle changes followed by the addition of one or more oral hypoglycemic agents, followed by the addition of/replacement by insulin. Given that diabetes is a progressive disease, the need for insulin in people with type 2 diabetes, will continue to rise. A team approach is also recommended; the person with diabetes is at the core of this team with the primary care physician, diabetes nurse educator, dietitian, diabetes and other specialists, and others also being members. The CDA Clinical Practice Guidelines recommend a A1c < 7.0%. Although A1c is considered to be the best measure of overall glycaemic control, blood or capillary glucose levels are still important to obtain in order to safely and effectively manage the doses of the oral hypoglycemic agents and insulin. This can best be done by having the person with diabetes perform self-monitoring of blood glucose (SMBG) at various times of the day.

There is now evidence in both type 1 as well as type 2 diabetes that improved glycaemic control will reduce the microvascular complications of diabetes[44]. An epidemiologic analysis of the UKPDS[45] showed that there is no threshold for benefit when it comes to glucose lowering, and that any reduction in A1c is associated with reduced risks for both micro- and macrovascular disease. In their analysis, every 1% decrease in A1c was associated with a 25% reduction in risk for myocardial infarction[45]. The ADVANCE study demonstrated a reduction of microvascular events with normalization of A1c to below 7.0%[4]. The ACCORD study which targeting aggressive normalization of A1c in patients with longstanding diabetes, found an elevated mortality in the intensive group which targeted normalization of blood gluocse[5]. The goal of diabetes intensification in this study is to reduce the risk for the complications of diabetes by controlling blood glucose safely.

### Rates of use of insulin and hypoglycemia, evidence from clinical trials

Studies of glycaemic control in people with type 1[46] and type 2[44] diabetes have shown improvements in complications such as the progression of kidney, eye and heart disease. Achieving control of blood glucose requires adherence to lifestyle changes as well as titration of medications, including the initiation of insulin therapy and the titration of the insulin dose. The intensive treatment group of the STENO-2 study led to over 50% being started on insulin with an average dose of 62 units per day[47]. Of interest, a greater proportion of the usual care group was started on insulin and there was a trend to higher doses. The glycaemic target in the intensive arm of the STENO-2 study was an A1c of 6.5%, compared to about 7.5% in the control arm. Despite lower blood glucose levels attained in the intensive treatment group, there were fewer major hypoglycemic complications (eg. impaired consciousness which required help from another person) in the intensive treatment compared to the control group (just under 1% vs. 2% per patient year respectively)[47]. In the UKPDS 33 study the rates of major hypoglycemic episodes per year were also relatively low, 0.7% with conventional treatment, 1.0% with chlorpropamide, 1.4% with glibenclamide, and 1.8% with insulin[48,49]. Fear of the complications of insulin therapy, particularly hypoglycemia, by both patients and health care workers, is one of the major barriers to implementing intensification of diabetes management. The ability to monitor blood glucose levels remotely in people who are having their diabetes medications adjusted may go a long way to providing the same kind of safety net for diabetes management that was the rationale for managing diabetes in the hospital setting years ago.

### The Battlefords Tribal Council Indian Health Services and approach to diabetes

The Battlefords Tribal Council Indian Health Services is a First Nations owned and operated Health Services organization established over 26 years ago. The Board of Directors consists of the seven Chiefs of the Battlefords Tribal Council First Nations. The population of each of the seven communities ranges from 500 to 700 people for a total population of 4,121 persons living on reserve[50]. North Battleford serves as the central base for the delivery of health services for the seven communities. All communities are within 90 kilometers of medical facilities. The culture is primarily Cree, and Cree and English languages predominate. A survey completed in 1999 found that diabetes, heart disease, cancer and high blood pressure were the top four most serious health-related problems for the community[50]. Diabetes was found in 22% of adults[51].

The Home and Community Care Diabetes Education Program has a ten year history and has been constantly evolving during that time. The Program encompasses all levels of prevention: primary, secondary and tertiary. Program staff includes one Home Health Aide and Registered Home Care Nurse in each community. A dietitian, diabetes nurse educators and exercise therapist serve all communities. Four of the staff members (1 dietitian and 3 nurses) have received Canadian certification as Diabetes Educators. All program development considers cultural appropriateness; in addition, nine staff members are fluent in the Cree language.

The Home Care team has always had a primary prevention component and this has been significantly augmented in the last three years through the Aboriginal Diabetes Initiative that has helped to increase community access to fresh fruits and vegetables through a Good Food Box program; a granting process to support community-based initiatives (walking groups, healthy snacks, cooking groups); a training program for staff who cook meals in the schools; and, a healthy lifestyles newsletter. Secondary prevention is addressed through diabetes education provided to people with diabetes and members of their family. Another component of client education is foot assessment and care; nurses hold regular foot care clinics. Prior to the DreamTel program, the Home Care team did not provide diabetes treatment or management. Instead, staff members referred clients to their family physician and advocated for treatment, with referral to specialists as needed. Prior to the DreamTel program, for the initiation of insulin in community members with type 2 diabetes, it was standard practice for primary care providers to arrange for admission to hospital for insulin initiation. Diabetes intensification was coordinated through the Diabetes Education Center at the Battlefords Hospital. However, its distance from people living on reserve with diabetes limited their access for repeated visits and ongoing education and treatment. At the time the DreamTel program was initiated, there were no diabetes specialists in Northern Saskatchewan, so that there was no backup for primary care providers. All of this added to the risk for clinical inertia for diabetes intensification and the initiation of insulin.

The ability to accurately monitor blood glucose results with self monitoring of blood glucose (SMBG) is pivotal to the success of managing and controlling blood glucose levels in people with diabetes on insulin. Glucose meters in current use require the user to record the displayed result into a logbook along with the timing of the reading. This allows cumulative readings to be analyzed for patterns. Barriers to health care workers receiving accurate logbooks include the ability and willingness of the person using the glucose meter to record the results accurately and to bring the logbook with them to appointments with health care providers. Evidence provided by glucose meters with memories has demonstrated that the recording of results into logbooks may not be accurate[52], even when glucose meters users know that their device is also recording the levels. Furthermore, health care providers only get access to the information in the logbook or that stored within the glucose meter during patient or client visits. This may lead to prolonged episodes of dysglycaemia before the client can receive medical attention. Bluetooth enabled glucose meters facilitate the downloading of the results to a secure electronic format available to health care providers via a web interface. This may help to eliminate the effects of geography and will allow the potential for more frequent health care monitoring of SMBG. The health care team can arrange for more frequent monitoring during titrations of drug therapies or the initiation of insulin. Patients with questions about their management will be able to initiate a health care provider review of their results by phone-call without generating a visit.

The ultimate goal of the DreamTel project is to develop a process for initiating insulin therapy in the community setting including intensification of diabetes management in a culturally appropriate manner. If successful, the legacy of the project will be a change in medical practice in the community with the introduction of a chronic disease management model for type 2 diabetes management, where primary care physicians can consult with the Home and Community Care team for initiation of insulin therapy in the community as well as intensification of diabetes therapy.

## Methods and design

The DreamTel project is designed to be carried out in 3 phases. In the first phase, the new technology will be introduced to volunteer subjects with uncontrolled type 2 diabetes. The second phase includes the development and implementation of a nurse directed medical management model for type 2 diabetes to allow a change in practice within the community. The goal is to allow primary care physicians in the community to refer patients to the Home Care team for initiation of insulin and diabetes intensification. This process involves health care provider education, the development of algorithm driven protocols based on clinical practice guidelines, patient education and disease measurement. In the third and final phase, subjects from the first phase who successfully used the device 80% of the time will be invited to participate in diabetes intensification with oversight from their primary care physicians.

### Phase 1: Introduce the Bluetooth-enabled glucose meters into the community

Experimental Design. The target populations for the DreamTel study are participants with Type 2 diabetes and uncontrolled blood glucose levels with A1c levels in excess of 7.0%. Fifty subjects are targeted in the pilot study. Clients willing to participate in the program who have uncontrolled diabetes based on A1c levels above target will receive information on the use of the Bluetooth enabled device and will be consented into the project. They will then have the device installed into their home and receive training on its use from the Home Care team. Technical support is available from the sponsor. For the first phase, lab testing for A1c takes place at initiation of the device and after three months. Subjects will be identified by their primary care physician or by the Home Care team as needing diabetes intensification. Thus they will constitute a convenience sample. Subjects will be trained in the use of the glucose meters but do not have to be taking insulin on enrollment. All will be trained on the Bluetooth technology. The LifeStat service is an Integrated Communications Technology to capture physiological data that is transmitted via Bluetooth to an aggregation device located at the point of care. The aggregation device can be a cell phone but in the DreamTel study due to insufficient local coverage a Bluetooth/Analog modem with the local telephone will be used. The data is then forwarded to a secure hosting center by standard telephone network. The information is then accessed by users with the appropriate credentials through a care giver portal.

All subjects receiving the device will have a multi-disciplinary team diabetes assessment with standard diabetes screening tests including a baseline A1c level and agree to follow-up lab work on a three-month basis throughout the duration of the study. Each study subject will receive training in how to use the device and will be instructed on how to indicate that a test run was a validation test or another person's sample. Subjects will be instructed to use the results of the testing to manage their diabetes according to the education they received in light of food intake (or lack of), physical activity and whether medication had been taken. The client will be taught how to use the device and how to demonstrate that they were able to accurately use it. Data forwarded to the web server will be displayed through the portal graphically and include summary statistics. Data can also be printed by the patient's health care providers and could became part of the client's medical record. All consenting subjects will be asked to notify their primary care physicians that they are using the new device during regularly scheduled visits. Their physicians will also receive information packages from the Home Care team on the study and will be invited to participate in local Continuing Health Education programs on the study and the technology used. To determine whether the installation of the LifeStat device linked to the patient's telephone line impacts on blood glucose control, the A1c level at baseline (prior to its installation) will be compared to the three-month value. Typically A1c levels remain stable over short periods of time, rising over a period of years[45].

To provide data on whether the blood glucose monitoring was before or after a meal, a teleprompt will be delivered to the client's phone. When a blood glucose result is received by the central server at SaskTel's central office in Saskatoon, Saskatchewan, an automatic teleprompt will call requesting a response as to the relationship between the test result and the last food intake. The teleprompt was developed in English and in Cree. Patients will be trained in its use. Each patient's impression of the teleprompt system will be addressed in questionnaires administered after the first 3 months of its use. Learning challenges will be collected as qualitative data to record the duration of time spent teaching and to record the challenges faced.

Another objective for the first three months of use is to compare data from three different sources; data transmitted by the device and stored on the web site, data stored on the device itself and the patient's log book, noting discordant values. Data is to be presented as the proportion of results recorded accurately. The frequency of monitoring based on data from the electronic output is to be assessed for the entire period.

### Phase 2: Development of a Chronic Disease Management model for initiation of insulin in the community and intensification of blood glucose control

The medication algorithm is broken into stages and can be found in Additional File 1: Initiation of metformin, initiation of sulphonylurea, initiation of thiozolidendiones and finally initiation of insulin followed by multidose insulin. A plan of management for diabetes intensification will be prepared for each patient based on their stage at their enrollment into phase 3, the treatment phase of the study. This plan will describe the dietary and lifestyle intervention they will receive as well as the planned stage for medication for the diabetes intensification. Prior to initiating each stage of medical therapy, a letter will be sent to the Primary Care physician including the results of the blood glucose monitoring. The letter will indicate the stage of diabetes intensification recommended and will include a standardized Home Care order set for that stage. The order sets were reviewed with some of the local primary care physicians during invited continuing education sessions about the DreamTel program. Once the Primary Care physician has reviewed this and signed the order set this stage of diabetes intensification will begin. When the stage of intensification of diabetes has been completed the Home Care team will notify the Primary Care physician. If the patient has achieved diabetes targets they will be maintained at this stage. If they have not achieved target then the orders for the next stage will be sent to the Primary Care physician and the patient assessed for the next stage.

Algorithms developed for diabetes management were developed based on clinical practice guidelines and reviewed with experts in diabetes management. For self monitoring of blood glucose[1], the goal is a minimum of four times daily, two days per week for those on or starting insulin. Medication will be introduced in a stepped fashion for those on diet therapy alone starting with metformin followed by a sulphonylurea (gliclazide, as it is better tolerated in patients that develop kidney disease than others)[53], thiazolidendiones (TZD's) will be added if sulphonylureas have failed. Pioglitazone (Actos) was chosen for use as it is a once daily preparation. This step was added prior to the controversy over rosiglitazone[54]. The algorithm for diabetes intensification to be carried out by the Home Care team was incorporated into a standardized order form for the primary care physician to approve. The study team in partnership with the primary care physician will be responsible for assessing contraindications to the algorithms.

The initiation of insulin is often frightening for patients and a program of education and an algorithm for the introduction of insulin were developed. At all times, the primary care physician will be kept in the loop as part of a management model. After Home Care visits, reports on study letterhead will be faxed or mailed to the primary care physician. In addition, a system of graded alerts developed by the Home Care team including the use of the existing Provincial emergency Health Hotline and medical backup will be used in the study.

The goal of this phase of the program is to facilitate a change of practice to improve diabetes management over the long term. Physicians and the Home Care team need to become integrated and systems developed to do this. The hope is that following the study, patients who require diabetes intensification will be identified by the physician, the Home Care team and possibly by patients themselves. The Primary Care Physician would then initiate the process by signing a completed Home Care referral form for the initiation of intensification of diabetes management. This will initiate a visit by the Home Care team (nurse or dietitian).

Following three months with the device, if patients are testing at 80% of the target for measurement, they will be enrolled in the next phase of the program and receive diabetes intensification therapy. At this point, following the first phase, patients willing to participate in the program will received a physical exam and assessment of their current diabetes status and medications with a specialist and a determination on which part of the diabetes intensification algorithm they are in.

In the Province of Saskatchewan there is a billing code for the supervision of diabetes intensification. Educational sessions for the primary care physicians will include the rationale for the DreamTel study, a review of the protocol itself and information on this billing code and its appropriate use for the management of these patients. It is a goal of the DreamTel study that the Primary Care physicians will be able to continue to work with the Home Care team for the intensification of diabetes management after the project has been completed.

Home care referral forms were developed for the primary care physicians. It was planned that if the primary care physician did not want the Home Care team to do the intensification of diabetes management according to the planned algorithm that the subjects would be followed as a control. If the primary care physician directs the Home Care team to manage the patients in a similar fashion, only using different medications from the algorithm, based on patient specific factors, these patients will remain in the study. Referrals to the local diabetes education centre will be tracked if any occur. The commencement date for intensification will be recorded. Intensification is defined as increasing the dosage of a medication, adding another medication or initializing insulin according the algorithm for intensification.

For clients on oral anti-hyperglycemic agents, designated RN(s) who are part of the Home Care team will adjust these medications based on the protocol. Off-standard situations will be reviewed with one of the specialist study physicians.

The study endocrinologist (RL) will regularly review the medication adjustments for randomly selected clients as a quality control procedure. The Diabetes Educators will be able to review off-standard situations with him for advice and support. The web-based glucose readings will foster this process as the Diabetes Educator and endocrinologist will be able to view the client records and adjustment concurrently via the Internet. Time has been set aside for this on one weekday morning each week by teleconference. Development of the nurse protocol for the proposed study is based on the adaptation of the provincial template by an experienced Certified Diabetes Nurse Educator.

### Phase 3: Achieve better control of blood glucose levels

Rationale. Based on the success of the DREAM study where a chronic disease management model resulted in improved blood pressure control[7], the DreamTel study hopes to demonstrate that a chonic disease management program can improve glycemic control in First Nations People with diabetes.

Experimental Design: The main intervention will be applied to the treatment group and will consist of diabetes management intensification including a stepped drug protocol algorithm for pharmacologic management of blood glucose including the addition of insulin when indicated, diet and lifestyle education, with the long term goal of achieving A1c levels of ≤ 7.0%. (see Additional File 1)

The study is designed as a pilot consisting of fifty subjects. Subjects entering this phase of the study will have type 2 diabetes and will have used the LifeStat blood glucose monitoring device for 3 months. Subjects will have completed at least 80% of all recommended glucose meters readings and will have been agreeable to intensification of diabetes according to the treatment algorithm. Diabetes intensification includes as needed, the initiation of oral hypoglycemic agents, the addition or increase of dosage of oral hypoglycemic agents and the initiation and dose titration of insulin. A1c levels will be drawn on a three-monthly basis. If fewer subjects than anticipated make it through phase 1 and are eligible for phase 3, additional subjects will not be recruited. As this is a pilot study, the reasons that fewer subjects than expected are able or willing to enroll into phase three will be explored.

All patient information specific to diabetes management from each Home Care visit to implement the intensification protocols will be forwarded to the patient's family physician. Follow-up with the patient's primary care physician will be requested by the Home Care team with each medication change to ensure that they are aware of the patient's new treatment status, and that it corresponds to their desired treatment goals for that patient. The Home Care team will be responsible for attempting to maintain ongoing contact with their clients if their clients accept that contact. Participants will be provided with snacks and meals as well as health-promotion materials part of current protocols. Additionally the study sponsor provided gifts such as t-shirts and mugs for study visits to see the study investigators, part of preexisting health promotional activities.

The initial assessment will include lab testing and historical information to address risk factors for cardiovascular-renal disease. The results of all testing will be forwarded to the primary care physician; subsequently, protocol-driven therapy for blood sugar control will begin. Medication doses and compliance will be closely monitored and prescriptions will be adjusted, as necessary. In the DREAM3 study, 45% of subjects had A1c levels ≥ 8.0%[7].

This investigation will be conducted in North Battleford, Saskatchewan. The DreamTel nurse coordinator will be based at the Battlefords Tribal Council Health Care services in North Battleford and will coordinate all aspects of the study under the supervision of Joan Wentworth. Dr. Lewanczuk will be responsible for the diabetes management algorithm. Data collection and management will be coordinated by Dr. Sheldon Tobe. A data clinical research assistant, based in Toronto, will be responsible for the monitoring, data collection and data entry. Statistical analysis will be performed at the Institute for Clinical Evaluation (ICES) in Toronto.

### Inclusion criteria

Type 2 Diabetes with baseline A1c ≥ 7.0% on diet or medical therapy with no history of diabetic ketoacidosis. Patients must be greater than or equal to 18 years of age. Written informed consent must be obtained prior to admission to this study.

### Analytic procedures and sample size calculation

This is a pilot study that will compare, in a before and after methodology, the intervention of diabetes intensification on the primary response variable of blood glucose level (A1c) measured at baseline and at 6 months. The pilot study will also determine the ability to enroll patients into the study and determine how many are able to maintain an 80% adherence rate to the use of the glucose meter four times daily at least twice weekly. This is also a goal of phase 3 of this study. See Additional file 2 for statistical design considerations.

Initiation of Diabetic Medication Treatment: The goal of Intensification therapy is A1c <7.0%. Patients in the usual care arm will continue to see their primary care physicians. Therapy in the treatment group will be initiated with the goal to bring the fasting plasma glucose ≤ 6 mmol/L and in insulin treated patients, pre-meal glucose concentrations of 4–7 mmol/L.

Safety and Interim Monitoring. Drug therapy may be interrupted at any time during the trial if further evidence for more appropriate drug therapy becomes available. All drugs used in this trial are available in regular clinical practice. Women of child-bearing potential must have a negative urine pregnancy test prior to receiving study medication. Patients will be asked for adverse events from the last visit related to hypoglycemic episodes and these data recorded. In the UKPDS 33 study the rates of major hypoglycemic episodes per year were 0.7% with conventional treatment, 1.0% with chlorpropamide, 1.4% with glibenclamide, and 1.8% with insulin [48,49]. Severe hypoglycemia defined as needing the assistance of another person, or coma, will be tracked, as will diary-recorded instances.

Case report forms, confidentiality, data security and monitoring. Case report forms will be completed by the study nurse in Saskatchewan and faxed to the coordinating centre in Toronto after all patient identifiers have been removed; data monitoring will be completed on each visit. The original forms will be kept on file at the Battlefords Tribal Council Indian Health Services. All data is owned in collaboration with the Battlefords Tribal Council Indian Health Services. All work for publication from this data will be shared with this group prior to submission and requests for aggregate data will be honored as quickly as resources permit. At all times the Investigators have the final responsibility for the accuracy and authenticity of all clinical data entered on the CRF's. The study will be conducted according to the Battlefords Tribal Council Indian Health Services Ethical Guidelines for Research and the principles of Ownership, Control, Access, Possession[55]. The Data Coordinator/Monitor will conduct site visits four times annually at a mutually agreed upon time for the purpose of auditing the study. The local investigator and clinical coordinator agree to allow the monitoring of the clinical supplies storage area and study documentation.

Approvals. The Battlefords Tribal Council Indian Health Services Inc has passed a Tribal Council Resolution approving the implementation of the DreamTel study. Ethics approval has been obtained from the Sunnybrook Health Sciences Centre and the BTC Research Ethics Boards. (see Additional file 3)

Study subjects will be followed from study entry until study completion (18 month study protocol from the start of phase 1). The primary outcome of this phase of the study is the A1c after 6 months. A1c results collected beyond this will be analyzed if there are sufficient subjects. Subjects may withdraw from the study at any time. Subjects who refuse or are unable to adopt the recommendations of diabetes intensification management will be followed in an intent-to-treat fashion.

## Discussion

Our long term goal is to achieve targets for blood glucose and blood pressure in people with diabetes as outlined by clinical practice guidelines[1] and thus to prevent target organ damage. The specific hypothesis behind the proposed research is that diabetes care in First Nations people can be improved by the involvement of the Home Care team with the patient, the primary care physician and the specialist in a chronic disease management program. The goal is to develop and implement a Chronic Disease Management Program for diabetes intensification which includes the four factors that have been shown to work when combined together; provider education, patient education, clinical practice guidelines and disease measurement [56]. In this study, diabetes intensification will be supported by emerging technology, specifically Bluetooth-enabled glucose meters and blood pressure monitors, capable of transmitting their data to a web environment in tabulated and graphical format available for remote viewing. This hypothesis is based on the following observations and evidence: First, despite current clinical practice guidelines and evidence-based practice by the medical community, including family physicians and the Home Care team, blood glucose control in general and in the First Nations community specifically is not meeting targets [1,57]. Poor control of blood glucose in type 2 diabetes dramatically increases the risk of heart disease, stroke, vascular limb loss, blindness, kidney disease and the need for dialysis. Control of blood glucose levels prevents these complications. Second, the intensification of diabetes control including the initiation of insulin is not easily done during a regular primary care visit. The lack of intensification of diabetes management in people with poorly controlled diabetes has been referred to as 'clinical inertia' [58]. While subjects referred to specialists have somewhat better control of diabetes, more than 50% still failed to have intensification of diabetes management within four months of a lab report showing a high level of A1c [58]. Diabetes specialists are not accessible to all primary care practitioners. Third, multidisciplinary care can overcome clinical inertia, even in difficult practice settings. We recently demonstrated, for intensification of blood pressure control, that a program delivered by the Home Care team could achieve target blood pressure in almost 50% of patients [7]. In addition, this program, which was closely linked with the primary care community and hypertension specialists, improved communication and health care provider satisfaction with the management of this condition. Fourth, the emerging technology of Bluetooth-enabled devices and secure web based monitoring logs allows health care providers for the first time to remotely monitor progress, providing a higher level of confidence in self blood glucose monitoring (SBGM) and blood pressure results. The specific aims are designed to provide a comprehensive assessment of the impact of the introduction of this program.

This study has limitations inherent to its design as a pilot and reliance on a convenience sample of subjects. Further, with a limited number of family physicians, it is possible that a change of practice may reduce the number of subjects with poorly controlled diabetes. This will also make a subsequent randomized study more difficult without some sort of cluster design at the level of the primary care physician or even community.

Diabetes is among the most debilitating of all chronic illness. First Nations people living on reserve have high prevalence rates of diabetes and those ill with this condition are then challenged by the long distances they must travel to receive specialized diabetes management. Furthermore, family physicians are now challenged by a lack of specialty care for complex diabetes management issues. We believe that the Home Care team can bridge these treatment gaps using available technology including remote specialty consultation when needed. Home Care nurse management of blood glucose control will lead to earlier identification of people who are at risk of the complications of high blood glucose and the Home Care multidisciplinary team will lead to greater control of blood glucose. Accomplishing the specific aims outlined in this proposal will provide the foundation required to roll out this methodology to rural and Northern communities.

## Competing interests

The study pilot has been funded by SaskTel. The funding body had no role in the design of the study or in the writing of this manuscript or decision to submit the manuscript for publication. They will also have no role in any aspect of the described data management, analysis or the reporting of study results. The authors declare that they have no competing interests.

## Authors' contributions

All the listed authors participated in the design and protocol development and assisted in drafting the manuscript. AK carried out the statistical analysis. All authors read and approved the final manuscript.

## Pre-publication history

The pre-publication history for this paper can be accessed here:



## Supplementary Material

Additional File 1 **DREAMTEL – Drug Algorithm for Intensification of Diabetes Management**. The protocols for intensifying diabetes management broken into 6 stages to allow each stage to stand alone as a standing order set. Stage 1: Introduction of metformin. Stage 2: Introduce: Gliclazide (Insulin Secretogogue – Sulfonylurea (SU)) and titrate OR Repaglinide (Gluconorm) and titrate. Stage 3: Insulin Sensitizer – Actos (Pioglitazone), Thiazolidinediones (TZD). Stage 4: Start hs long acting insulin. Stage 5: Adding a second dose of basal insulin. Stage 6: Converting Split Dose Basal Insulin to Multiple Doses of Rapid Acting with NPH late evening. Guide for using oral medications for diabetes intensification using the stage protocol for people not on insulin. Click here for file

Additional File 2 **DreamTel Statistical Plans for Subsequent Study**. Statistical plan for subsequent research. Click here for file

Additional File 3 **Battlefords Tribal Council Health Services Ethical Guidelines for Research Purpose**. A statement outlining the principles of conducting clinical research with Fist Nations people of the Battlefords Tribal Council. Click here for file
