# Supplementary material for: DreamTel; Diabetes risk evaluation and management tele-monitoring study protocol
Source: BMC Endocr Disord. 2009 May 9;9:13. doi: 10.1186/1472-6823-9-13 (PMC2689225; doi:10.1186/1472-6823-9-13)
Supplement: Additional File 1 — DREAMTEL – Drug Algorithm for Intensification of Diabetes Management. The protocols for intensifying diabetes management broken into 6 stages to allow each stage to stand alone as a standing order set. Stage 1: Introduction of metformin. Stage 2: Introduce: Gliclazide (Insulin Secretogogue – Sulfonylurea (SU)) and titrate OR Repaglinide (Gluconorm) and titrate. Stage 3: Insulin Sensitizer – Actos (Pioglitazone), Thiazolidinediones (TZD). Stage 4: Start hs long acting insulin. Stage 5: Adding a second dose of basal insulin. Stage 6: Converting Split Dose Basal Insulin to Multiple Doses of Rapid Acting with NPH late evening. Guide for using oral medications for diabetes intensification using the stage protocol for people not on insulin. [file 1472-6823-9-13-S1.doc]

Additional File 1

# DREAMTEL – Drug Algorithms for Intensification of Diabetes Management

# Stages

Description: The protocols for intensifying diabetes management broken into 6 stages to allow each stage to stand alone as a standing order set.

Stage 1: Introduction of metformin

Stage 2: Introduce: Gliclazide (Insulin Secretogogue – Sulfonylurea (SU)) and titrate OR Repaglinide (Gluconorm) and titrate.

Stage 3: Insulin Sensitizer – Actos (Pioglitazone), Thiazolidinediones (TZD)

Stage 4: Start hs long acting insulin

Stage 5: Adding a second dose of basal insulin

Stage 6: Converting Split Dose Basal Insulin to Multiple Doses of Rapid Acting with NPH late evening

Guide for using oral medications for diabetes intensification using the stage protocol for people not on insulin.

**Client not using any oral medications, not on insulin:**

1. Introduce Metformin, titrate
2. Introduce Gliclazide or Repaglinide, titrate
3. Introduce Actos, titrate

**Client already on one or more oral medications:**

Review pattern of blood glucose readings, review usual eating and activity habits and guide and choose from the following options:

- 1. Go to Stage 1, introduce Metformin
  2. Go to Stage 2, introduce Gliclazide or Repaglinide
  3. Go to Stage 3, introduce Actos
  4. Go to Stage 4, introduce insulin

**Introduction of insulin and adjustments:**

1. Introduce late evening insulin and titrate
   - Review pattern of glucose readings and move to stages 5 or 6 depending on the blood glucose pattern and client input
2. Add a second basal insulin to be taken fasting
3. Add rapid acting insulin pre-meal – the number of times will vary with the usual eating and activity habits and the pattern of the blood glucose readings

Exception:

If client is elderly or unable to use any of the above regimens, could consider pre-mixed insulin such as 30/70 given od or more likely bid

**Client is already using insulin:**

Review the pattern of blood glucose readings and discuss with the client and consider usual eating and activity habits, choose from the following options:

- - Adjust existing insulin regimen according to the pattern of blood glucose readings to achieve targets pre and post meals
  - Split pre-mixed insulin into its individual insulins to titrate doses according to the pattern of blood glucose readings
  - Go to Stage 4 (may move pre-supper intermediate insulin to late evening)
  - Go to Stage 5 to add or adjust fasting basal insulin
  - Go to Stage 6 to add or adjust pre-meal rapid acting insulin
  - Convert from bid insulin to multiple injection system, Stage 6

**Use of extended long acting insulin [Lantus or Levemir]**

At present these insulins are only available for type 1 persons. Exception status may be possible or be changed over the course of the intensification period to make these insulins an option.

**Targets for Blood Glucose Control:**

- 4-7 mmol before meals; 4-6 mmol (optimal)
- 5-10 mmol 1½ -2hr pc; 5-8 mmol (optimal)
- A1C ≤ 7%;

# Procedures for Using the Protocol:

**Documentation**:

All medications, changes in doses and client side effects with any medication will be recorded on the medication case record. Documentation will also indicate contraindications to the use of any medication; client refusal to use a medication or reasons why a medication was not tolerated by a client.

**Initial Assessment**:

The initial assessment for each client will include

- a history for current and past diabetes medications (medication, dose, frequency, prescribed versus actual dose/frequency)
- review of symptoms related to medications, including hypoglycemia
- review of relevant lab results for each medication
- summary of any medication contraindications
- documentation of the client’s current status on the protocol steps

The initial client medications will not be changed unless there is a contraindication to the use of a medication or the change is part of the medication management intensification. (Appendix A)

**Medication Protocol Steps**:

Advancement in medication stages will be based on

- ongoing assessment of client’s self management (healthy eating, physical activity, medication use and achievement of target blood glucose levels)
- contraindications to use of any of the suggested medication
- client readiness to change medication (type, dose or frequency)

The advancement in the medication stages will be individualized and “off standard” situations will be discussed with the family physician, the consulting endocrinologist or both.

All changes in the medication step will be initiated with a signed physician order outlining the limits of the medication adjustment. The order will include permission to discontinue a medication in emergency circumstances without consulting a physician.

All medication changes will be recorded on the LifeStat electronic logbook and in the case report. The family physician will receive a copy of all medication changes.

A summary of the clients’ stage and progress will be sent to the family physician after each A1C is taken or more frequently, if needed.

# Stage 1

Introduce: Metformin (Biguanide) and titrate

Who?

- Client with type 2 DM on no oral anti-hyperglycemic medications or
- On a Sulfonylurea or TZD, not on Metformin,
- Creatinine ≤ 150
- A1C > 7.0

# Steps

1. Client education
   1. Ensure client is using glucose meter correctly
   2. Educate client about Metformin
2. Medication initiation and adjustment
   1. Start metformin 250 mg bid x 7 days
   2. Increase to 500 mg bid x 3 weeks
   3. Increase to 1000 mg q am and 500 mg q pm x 7 days
   4. Increase to 1000 mg bid
3. Document if client is experiencing GI disturbance. If so, reduce dosage to last tolerated stage. Check with client 2-3 weeks after, if no symptoms, continue to increase to step 6 (1000 mg bid).
4. At 12 weeks after initiation of Metformin, repeat A1C
5. If A1C >7.0, review blood glucose pattern, eating and activity habits and go to Stage 2, if appropriate.

[NOTE, if pre-meal glucose readings are at target, but A1C remains > 7.0, ask client to check blood glucose pc meals]

# Stage 2

Introduce: Gliclazide (Insulin Secretogogue – Sulfonylurea (SU)) and titrate OR Repaglinide (Gluconorm) and titrate.

Assess client’s pattern of blood glucose readings, usual eating and activity habits to ensure best application of protocol. Consult with specialist if client assessment indicates modification of the protocol may be needed.

Who?

- Stage 1 A1C > 7.0 OR on Metformin + TZD previously, without control on stable dose (do not increase Metformin at this stage, as client may have had previous GI disturbance)

# Steps for Gliclazide

1. Client education:
   1. Ensure client using glucose meter correctly
   2. Educate client about Gliclazide including symptoms, treatment and prevention of hypoglycemia. Advise client to check blood glucose, if possible, when symptoms of hypoglycemia occur.
2. Medication initiation and adjustment
   1. Start Gliclazide MR 30 mg po od before first meal of the day
   2. 2-3 weeks later if FBG ≥ 7.0 and no unexplained hypoglycemia, increase to 60 mg od
   3. 2-3 weeks later if FBG ≥ 7.0 and no unexplained hypoglycemia, increase to 90 mg od
   4. 2-3 weeks later if FBG ≥ 7.0 and no unexplained hypoglycemia, increase to 120 mg od
3. At 12 weeks after initiation of Gliclazide, repeat A1C
4. If A1C > 7.0, review blood glucose pattern, eating and activity habits and go to Stage 3, in appropriate.

[NOTE, if pre-meal glucose readings are at target, but A1C remains > 7.0, ask client to check blood glucose pc meals]

**Steps for Repaglinide**

- Recommended for clients whose meal times and/or frequency are irregular.
- Dose can be varied with carbohydrate counting if client is prepared to do this.
- Client must also be able to understand the need to omit Repaglinide if no carbohydrate is eaten at a meal.
- Introduction will be based on the pattern of blood glucose readings and it may be taken with 1-3 meals per day.

1. Client education
   1. Ensure client using glucose meter correctly
   2. Educate client about Repaglinide including symptoms, treatment and prevention of hypoglycemia. Advise client to check blood glucose, if possible, when symptoms of hypoglycemia occur.
2. Medication initiation and adjustment
   1. Start Repaglinide 1-3 times per day depending on the pattern of pre and post meal blood glucose readings
   2. Start 0.5 mg with each meal if no previous treatment or A1C < 8%
   3. Start 1 mg with each meal if on other oral meds (Metformin, TZD) or A1C > 8% - in deciding dose also consider level and pattern of glucose readings
   4. Increase dose based on the pattern of blood glucose readings – may not use the same dose for all meals and/or may vary with carbohydrate intake
   5. If starting dose is 0.5 mg increase to 1 mg, then increase by 1 mg increments
   6. If starting dose is 1 mg, increase by 1 mg increments
   7. Maximum daily dose should not exceed 16 mg, with a maximum of 4 mg per meal
3. At 12 weeks after initiation of Repaglinide, repeat A1C
4. If A1C > 7.0, review blood glucose pattern, eating and activity habits and go to Stage 3, if appropriate.

[NOTE, if pre-meal glucose readings are at target, but A1C remains > 7.0, ask client to check blood glucose pc meals]

# Stage 3

Insulin Sensitizer – Actos (Pioglitazone), Thiazolidinediones (TZD)

A1C > 7.0

Who?

- Stage 2 uncontrolled or
- On Metformin and Sulfonylurea previously without control or
- On Metformin and did not tolerate a Sulfonylurea

# Steps

1. Client education:
   1. Ensure client is using glucose meter correctly
   2. Educate client about Actos including symptoms of shortness of breath, ankle swelling. Assess current status re shortness of breath and edema as a baseline.
2. Medication initiation and adjustment
   1. Start Actos 30 mg po od
   2. Weigh client and assess for edema in two weeks. With symptoms or a weight gain of 5 kg or more, consult with family physician or endocrinologist. HCN may discontinue medication while waiting for consultation.
   3. Reassess client at any time if edema worsens
3. At 12 weeks after initiation of Actos, repeat A1C, weight and ALT (liver function)
4. If A1C >7.0, review blood glucose pattern, eating and activity habits and go to Stage 4, if appropriate.

[NOTE, if pre-meal glucose readings are at target, but A1C remains > 7.0, ask client to check blood glucose pc meals]

# Stage 4

Start hs long acting Insulin

Who?

A1C > 7.0

- On Metformin + SU + TZD uncontrolled or
- Not able to tolerate any of the previous stages or
- Referred on any oral medication for insulin initiation

# Steps

1. Client education:
   1. Ensure client is using glucose meter correctly
   2. Discuss the time action of NPH given in the late evening. Insulin should be taken in the late evening, + one hour of a pre-determined time. If client has irregular bedtime, choose a late evening time in consultation with the client.
   3. Practice insulin measurement (pen or syringe) and injection technique
   4. Teach storage of insulin
   5. Teach role of self glucose meter monitoring and insulin dosing
   6. Teach symptoms, treatment and prevention of hypoglycemia. Advise client to check blood glucose, if possible, when symptoms of hypoglycemia occur.
2. Medication initiation and adjustment
   1. If previously on oral anti-hyperglycemic medications, continue these during insulin initiation unless contraindicated by the pattern of blood glucose readings.
   2. Start Novolin NPH 10 units s/c at a set late evening time (+ one hour)
   3. Adjust insulin using results using fasting blood glucose results. Obtain 3 readings between each adjustment. Also review pattern of blood glucose readings at other times of the day.
      1. If fasting blood glucose is not decreased from pre-insulin level, increase by insulin by 4 units
      2. If fasting blood glucose is decreased from pre-insulin level, increase by 2 unit increments
   4. If FBS >7 .0 mmol/L continue to increase to a maximum of 30 units at HS
   5. If daytime blood glucose readings are at target, consider that the dose of sulfonylurea may need to be decreased to prevent hypoglycemia
3. See client twice weekly (or daily M-F if necessary) until he/she is confident with managing insulin regimen, then weekly for insulin adjustment.

- 1. Depending on the client circumstances, the HCN may be able to review blood glucose readings on-line and discuss changes with the client by telephone.

1. At 12 weeks after initiation of insulin, repeat A1C
2. Management of hypoglycemia:
   1. Review circumstances of hypoglycemia with client to determine possible causes. Ask client if blood glucose was done in relation to symptoms.
   2. If unexplained hypoglycemia occurs during the night or early morning (fasting), reduce late evening NPH insulin
      1. By 10% (minimum of 2 units) for readings < 4.0 mmol.
      2. By 20% (minimum of 4 units) for readings < 3.0 mmol.
   3. If daytime unexplained hypoglycemia occurs, reduce sulfonylurea by 50%, for further episodes discontinue
   4. Discuss off-standard situations with endocrinologist. HCN may make medication reductions prior to discussion with medical specialist
   5. If insulin or oral medication are reduced/discontinued, wait one week before making any increases and, if needed, discuss with endocrinologist

## Stage 5

Adding a Second Dose of Basal Insulin

Who? Stage 4 on Insulin – any dose of insulin and A1C > 7.0 and daytime blood glucose remains above target.

# Steps

1. If previously on oral anti-hyperglycemic medications, continue these during initiation of second basal insulin unless contraindicated by the pattern of blood glucose readings.
2. Arrange for a 0200-0300 a.m. glucose meter reading, if > 7.0 proceed. If < 7.0 review with Diabetes Specialist at weekly conference
3. Review the current pattern of blood glucose readings and the client’s usual eating and activity habits to consider the potential impact of changing insulin regiment.
   1. Note some clients may skip this Stage and move to Stage 6, depending on the pattern of blood glucose readings.
4. Client education:
   1. Ensure client is using glucose meter correctly
   2. Discuss the time action of NPH given in the morning, fasting. Insulin should be taken in the morning, + one hour of a pre-determined time. If client has irregular waking time, choose a morning time in consultation with the client.
   3. Review/teach insulin measurement (pen or syringe) and injection technique
   4. Review/teach storage of insulin
   5. Review/teach role of self glucose meter monitoring and insulin dosing
   6. Review/teach symptoms, treatment and prevention of hypoglycemia. Advise client to check blood glucose, if possible, when symptoms of hypoglycemia occur.
5. Medication initiation and adjustment
   1. Start fasting insulin with Novolin NPH 5 units
   2. Review the pattern of blood glucose readings. Adjust fasting NPH based on pre-supper readings. Obtain 3 readings between each adjustment
      1. Increase fasting NPH insulin by 2 units, if 3 pre-supper tests are > 7.0.
   3. As the daytime blood glucose improves, if fasting blood glucose drops below 7.0, may need to decrease the late evening NPH.
   4. Increase fasting insulin to maximum of 15 units if client > 90 kg or 20 units if client > 100 kg
6. Visit client 1-2 times per week until he/she is confident with the second dose of insulin. Then contact weekly for insulin adjustment based on blood glucose results.
   1. Depending on the client circumstances, the HCN may be able to review blood glucose readings on-line and discuss changes with the client by telephone.
7. At 12 weeks after initiation of morning NPH, repeat A1C.
   1. If A1C > 7.0, review with Diabetes Specialist at weekly conference.
8. Management of hypoglycemia:
   1. Review circumstances of hypoglycemia with client to determine possible causes. Ask client if blood glucose was done in relation to symptoms.
   2. If unexplained hypoglycemia occurs during the night or early morning (fasting), reduce late evening NPH insulin
      1. By 10% (minimum of 2 units) for readings < 4.0 mmol.
      2. By 20% (minimum of 4 units) for readings < 3.0 mmol.
   3. If daytime unexplained hypoglycemia occurs about 12 hours after morning insulin is taken, reduce morning NPH insulin
      1. By 10% (minimum of 2 units) for readings < 4.0 mmol.
      2. By 20% (minimum of 4 units) for readings < 3.0 mmol.
   4. If unexplained hypoglycemia does not occur in relation to peak action time of insulins, reduce sulfonylurea by 50%, for further episodes discontinue
   5. Discuss off-standard situations with endocrinologist. HCN may make medication reductions prior to discussion with medical specialist
   6. If insulin or oral medication is reduced/discontinued, wait one week before making any increases and, if needed, discuss with endocrinologist
9. At any step, may stop titration and review with family physician or specialist.

**Stage 6**

# Converting Split Dose Basal Insulin to Multiple Doses of Rapid Acting with NPH late evening

Who?

- Stage 5 and A1C not controlled
- On split dose insulin with either, only long acting basal twice daily, or any other regimen (such as bid short and intermediate acting or pre-mixed insulin)
- A1C > 7.0
- Client willing to take rapid acting insulin prior to meals

# Steps

1. Arrange for a 0200-0300 a.m. glucose meter reading, if > 7.0 proceed. If < 7.0 review with Diabetes Specialist at weekly conference
2. Review the current pattern of blood glucose readings and the client’s usual eating and activity habits to consider the potential impact of changing insulin regiment.
3. Client education:
   1. Ensure client is using glucose meter correctly
   2. Discuss the time action of Novorapid given pre-meals, 1-3 times per day. Insulin should be taken 0-5 minutes prior to eating. Ensure clients knows he/she must eat immediately after taking rapid acting insulin.
   3. Review/teach insulin measurement (pen or syringe) and injection technique
   4. Review/teach storage of insulin
   5. Review/teach role of self glucose meter monitoring and insulin dosing
   6. Review/teach symptoms, treatment and prevention of hypoglycemia. Advise client to check blood glucose, if possible, when symptoms of hypoglycemia occur.
4. Medication initiation and adjustment
   1. Determine dose of basal insulin to be given in the late evening:
      1. Determine total daily dose (TDD) of insulin
      2. Use 40% of this dose as the initial basal dose of Novolin NPH
   2. Determine dose of pre-meal rapid acting insulin to be given 0-5 minutes prior to eating
      1. Determine total daily dose (TDD) of insulin
      2. Use 60% as the total pre-meal rapid acting insulin
      3. Divide the total pre-meal insulin amount by 3 to determine the amount to be given prior to each meal
         - Review this dose within the context of the number meals eating per day – the client may take rapid acting insulin 1-3 times per day
         - Review this dose within the context of the usual amount of carbohydrate eaten at each meal to help anticipate fine tuning of the doses
   3. Stop sulfonylurea (SU) – oral agent.
   4. Insulin adjustments to be made after 3 tests done at a relevant time for each insulin:
      1. Adjust late evening NPH using fasting reading
      2. Adjust pre-meal insulin based on blood glucose reading two hours post meal
   5. Determine need for a second basal insulin by pre-supper blood glucose reading – discuss with diabetes specialist
   6. Increase each insulin by 10% of dose, as appropriate based on the blood glucose pattern until targets are achieved
5. After 2 weeks of readings on new regimen, review with diabetes specialist at weekly conference to adjust dosing to glucose patterns
6. Management of hypoglycemia:
   1. Review circumstances of hypoglycemia with client to determine possible causes. Ask client if blood glucose was done in relation to symptoms.
   2. If unexplained hypoglycemia occurs during the night or early morning (fasting), reduce late evening NPH insulin
      1. By 10% (minimum of 2 units) for readings < 4.0 mmol.
      2. By 20% (minimum of 4 units) for readings < 3.0 mmol.
   3. If daytime unexplained hypoglycemia occurs, reduce the rapid acting insulin given immediately prior to the time of the hypoglycemia
      1. By 10% (minimum of 2 units) for readings < 4.0 mmol.
      2. By 20% (minimum of 4 units) for readings < 3.0 mmol.
   4. If an insulin is reduced/discontinued, wait one week before making any increases and, if needed, discuss with endocrinologist
7. Discuss off-standard situations with endocrinologist. HCN may make medication reductions prior to discussion with medical specialist
8. Consider the option of using pre-meal rapid acting insulin based on carbohydrate counting and a correction dose for blood glucose readings above target. This system will be tailored for each individual in consultation with the dietitian and diabetes specialist.
9. At any step, may stop titration and review with family physician or specialist.

Guide for Using Oral Medications Using the Stage Protocol for Persons NOT on insulin

1. Review usual eating and activity habits
2. Review medical and medication history to exclude any of the medications – document exclusions
3. Review labs to exclude any of the medications – document exclusions
4. Review pattern of recent blood glucose readings
5. If client is on maximum [or close to] of oral medications he/she is able to tolerate, move to stage 4
6. If longstanding diabetes, already on any oral medications, and A1C > 8.5%, consider moving immediately without adding additional oral medications, with client agreement, to stage 4

| BASELINE ->  STAGE ↓ | No oral meds | Metformin  Only | Metformin +  Sulfonylurea | Metformin +  TZD | Sulfonylurea only | TZD only | Sulfonylurea and TZD |
| --- | --- | --- | --- | --- | --- | --- | --- |
| Stage 1 | Metformin | X | X | X | Metformin | Metformin | Metformin |
| Stage 2 | Gliclazide or Repaglinide | Gliclazide or Repaglinide | X | Gliclazide or Repaglinide | X | Gliclazide or Repaglinide | Gliclazide or Repaglinide |
| Stage 3 | Actos | Actos | Actos | X | Actos | X | X |
| Stage 4 | Insulin | Insulin | Insulin | Insulin | Insulin | Insulin | Insulin |

NOTES:

- Use medication protocol to determine start doses and titration
- If client is already on Glyburide (Sulfonylurea) do not change unless issues with hypoglycemia or elderly (to reduce the risk of hypoglycemia)
- If client is already on Avandia (TZD) do not change
