# Supplementary material for: DreamTel; Diabetes risk evaluation and management tele-monitoring study protocol
Source: BMC Endocr Disord. 2009 May 9;9:13. doi: 10.1186/1472-6823-9-13 (PMC2689225; doi:10.1186/1472-6823-9-13)
Supplement: Additional File 2 — DreamTel Statistical Plans for Subsequent Study. Statistical plan for subsequent research. [file 1472-6823-9-13-S2.doc]

Additional File 2

DreamTel Statistical Plans for Subsequent Study.

Ultimately the goal of the pilot is to lead to a randomized controlled study. The appropriate statistical device for the randomized design would be a two factor (Treatment x Time) repeated measures analysis of variance (RANOVA). The focus of analysis will be the detection of a significant treatment by time interaction. A natural log transformation of all A1C scores will be applied to normalize the data prior to (RANOVA). The following assumptions have been made to estimate sample size required: the effect size of the interaction was set to slightly smaller absolute A1C decreases (a drop of 1.5 units for stepped drug vs 0.5 units for standard treatment) than those published by Aubert et al, (A drop of 1.7 units for treatment vs 0.6 units for control) in a very similar study [1]. Menard also found a similar reduction in A1c levels with intensive multi-therapy with approximately a 1.5 unit drop of A1c in the treatment group and 0.5 in the control group[2]. The common standard deviation for A1c for this study is taken from the standard treatment arm of the Dream 3 study, the most appropriate estimate for this DreamTel study as it is in the same population [3]. The mean in DREAM 3 for those with A1c >8.0% was 9.62%, SD 1.8. A reduction in A1c in the control group (Halo effect) is also expected in DreamTel. Setting alpha to .05 and the within subject A1C correlation to a standard .5, a study with 48 subjects per treatment group will provide RANOVA with 80% power to detect a significant Treatment x Time interaction representing a moderate effect size of .29 of major clinical importance[4]. In the randomized controlled study, the enrollment goal would be increased by 10%, the predicted rate of dropouts over 1 year in DREAM 3, therefore 96/0.85 =113 would be needed.

Reference List

1. Aubert RE, Herman WH, Waters J, Moore W, Sutton D, Peterson BL *et al*.: **Nurse case management to improve glycemic control in diabetic patients in a health maintenance organization. A randomized, controlled trial.** *Ann Intern Med* 1998, **129:** 605-612.

2. Menard J, Payette H, Baillargeon JP, Maheux P, Lepage S, Tessier D *et al*.: **Efficacy of intensive multitherapy for patients with type 2 diabetes mellitus: a randomized controlled trial.** *CMAJ* 2005, **173:** 1457-1466.

3. Tobe SW, Pylypchuk G, Wentworth J, Kiss A, Szalai JP, Perkins N *et al*.: **Effect of nurse-directed hypertension treatment among First Nations people with existing hypertension and diabetes mellitus: the Diabetes Risk Evaluation and Microalbuminuria (DREAM 3) randomized controlled trial.** *CMAJ* 2006, **174:** 1267-1271.

4. Hintze J: *PASS 2000: Power analysis and sample size for Windows*. Kaysville, Utah: NCSS; 2000.
