# Supplementary material for: DreamTel; Diabetes risk evaluation and management tele-monitoring study protocol
Source: BMC Endocr Disord. 2009 May 9;9:13. doi: 10.1186/1472-6823-9-13 (PMC2689225; doi:10.1186/1472-6823-9-13)
Supplement: Additional File 3 — Battlefords Tribal Council Health Services Ethical Guidelines for Research Purpose. A statement outlining the principles of conducting clinical research with Fist Nations people of the Battlefords Tribal Council. [file 1472-6823-9-13-S3.doc]

Additional File 3

Battlefords Tribal Council Indian Health Services Ethical Guidelines for Research

Purpose

These guidelines have been developed to help ensure that, in all research in which Battlefords Tribal Council Indian Health Services involved is culturally appropriate, relevant to the community needs and respect is given to the cultures, languages, knowledge and values of Aboriginal peoples, and to the standards used by Aboriginal peoples to legitimate knowledge.

These guidelines represent the standard of "best practice" adopted by BTC Indian Health Services.

## Principles

Aboriginal peoples have distinctive perspectives and understandings, derived from their cultures and histories and embodied in Aboriginal languages. Research that has Aboriginal experience as its subject matter must reflect these perspectives and understandings.

Board of Directors must provide written approval for any research which is undertaken in collaboration with Battlefords Tribal Council Indian Health Services within the member bands.

The community must be involved as a full partner in all aspects of research. Continuous consultation and collaboration should characterize the partnership.

Researchers have an obligation to understand and observe the protocol concerning communications within any Aboriginal community.

Capacity is to be developed within the Battlefords Tribal Council Indian Health Services staff so that when the research is completed there will be increased ability in research knowledge and skills left within the organization and the communities. Battlefords Tribal Council Indian Health Services staff should be involved in aspects of research design, continuing education and ongoing support related to the research focus.

Researchers have an obligation to observe ethical and professional practices relevant to their respective disciplines.

BTC Indian Health Services and its researchers undertake to accord fair treatment to all persons participating in research.

# Guidelines

## Aboriginal Knowledge

In all research involving BTC Indian Health Services, researchers shall conscientiously address themselves to the following questions:

- Are there perspectives on the subject of inquiry that are distinctively Aboriginal?
- What Aboriginal sources are appropriate to shed light on those perspectives?
- Is proficiency in an Aboriginal language required to explore these perspectives and sources?
- Are there particular protocols or approaches required to access the relevant

knowledge?

- Does Aboriginal knowledge challenge in any way assumptions brought to the

subject from previous research?

- How will Aboriginal knowledge or perspectives portrayed in research products be validated?

## Consent

Informed consent shall be obtained from all persons and groups participating in research. Such consent may be given by individuals whose personal experience is being portrayed, by groups in assembly, or by authorized representatives of communities or organizations.

Consent should ordinarily be obtained in writing. Where this is not practical, the procedures used in obtaining consent should be recorded.

Individuals or groups participating in research shall be provided with information about the purpose and nature of the research activities, including expected benefits and risks.

No pressure shall be applied to induce participation in research.

Participants should be informed that they are free to withdraw from the research at any time.

Participants should be informed of the degree of confidentiality that will be maintained in the study.

Informed consent of parents or guardian and, where practical, of children should be obtained in research involving children.

## Collaborative Research

In studies located principally in Aboriginal communities, researchers shall establish collaborative procedures to enable community representatives to participate in the planning, execution and evaluation of research results.

In studies carried out in the general community that are likely to affect particular Aboriginal communities, consultation on planning, execution and evaluation of results shall be sought through appropriate Aboriginal bodies.

In community‑based studies, researchers shall ensure that a representative cross‑section of community experiences and perceptions is included.

The convening of advisory groups to provide guidance on the conduct of research shall not pre‑empt the procedures laid down in this part but shall supplement them.

The following are key points in research collaboration:

- A common vision must be developed amongst all stakeholders and include, as the ultimate outcome, the betterment of the health of Battlefords Tribal Council Indian Health Services communities and their members. The "driver" for action must derive from communities and their needs, not research and its protocols.
- Researchers and their participating colleagues will need to invest the necessary time and learning to develop relationships with: BTC Indian Health Services organization, Leadership both formal and informal, staff and community members so that community culture and values are respected and honored
- As relationships are developed, the decision-making processes must be collaborative for all aspects of the research including proposal and protocol development, implementation, data collection, reporting and use of results.

Community Involvement in Research

BTC Indian Health Services will create a community-based research advisory committee to review specific research projects. This committee will have community‑level membership representing all the communities. Staff members may be part of the committee, but the core of the membership will be from the communities. The major role of the committee will be to learn about and understand the proposed research and then give advice to researchers and staff members to ensure that community needs, values and culture are respected. All potential researchers will be expected to meet and talk with the committee, perhaps at several points in a study. The committee members have the potential to become champions and advocates for research in their own communities. In addition, researchers will need to talk directly with communities about proposed studies.

## Review Procedures

Review of research results shall be solicited both in the Aboriginal community and in the scholarly community prior to publication.

## Access to Research Results

- BTC Indian Health Services shall maintain a policy of open public access to final reports of research activities.
- Reports may be circulated in draft form, where scholarly and Aboriginal community response at this stage is deemed useful for Commission purposes.
- Research reports or parts thereof shall not be published where there are reasonable grounds for thinking that publication will violate the privacy of individuals or cause significant harm to participating Aboriginal communities or organizations.
- Results of community research shall be distributed as widely as possible within participating communities, and reasonable efforts shall be made to present results in non‑technical language and Aboriginal languages where appropriate.

## Community Benefit

In setting research priorities and objectives for community‑based research, BTC Indian Health Services and the researchers it engages shall give serious and due consideration to the benefit of the community concerned.

In assessing community benefit, regard shall be given to the widest possible range of community interests, whether the groups in question be Aboriginal or non‑Aboriginal, and also to the impact of research at the local, regional or national level. Wherever possible, conflicts between interests within the community should be identified and resolved in advance of commencing the project. Researchers should be equipped to draw on a range of problem‑solving strategies to resolve such conflicts as may arise in the course of research.

Whenever possible research should support the transfer of skills to individuals and increase the capacity of the community to manage its own research.

## Implementation

These guidelines shall be included in all research contracts with individuals, groups, agencies, organizations and communities conducting research sponsored by BTC Indian Health Services.

It shall be the responsibility, in the first instance, of all researchers to observe these guidelines conscientiously. It shall be the responsibility, in ascending order, of research managers, the Co‑Directors of Research, and BTC Indian Health Services itself to monitor the implementation of the guidelines and to make decisions regarding their interpretation and application.

Where, in the opinion of the researcher or the research manager, the nature of the research or local circumstances make these guidelines or any part of them inapplicable, such exception shall be reported to BTC Indian Health Services through the Co‑Directors of Research, and the exception shall be noted in the research contract or contract amendments as well as in any publication resulting from the research.

#### Process for Approval of Health Research in Battlefords Tribal Council Indian Health Services Communities

The researcher and the community need to meet for the purpose of discussion and approval of the research ideas and the protocol involved.

- A written request is submitted to the Executive Director of Battlefords Tribal Council Indian Health Services to request a meeting to discuss approval of a research idea and the protocol involved. The written request will include a summary description of the proposed research, a time frame for research, and the proposed protocol. The executive director will review the request in terms whether it fits within the mandate of Battlefords Tribal Council Indian Health Services. If it does, she will distribute to the appropriate program director(s) for review.
- The executive director and the appropriate program staff will review the request in terms of:
- Cultural appropriateness
- Compatibility with goals and objectives of Battlefords Tribal Council Indian Health Services
- Workload manageability
- Benefits to the community members
- Time Line appropriateness
- Suggested modifications to research request.
- If the research meets the requirements but further input and suggestions are needed, the Battlefords Tribal Council Indian Health Services staff will meeting with the researchers to discuss modifications to the proposed research.
- Once there is agreement to support the research from the program and organization prospective, the researcher will be asked to meet with the community advisory committee to explain the project and receive further guidance regarding cultural and community appropriateness. Once the Community Advisory Committee support the research idea it is presented to the Board of Directors of Battlefords Tribal Council Indian Health Services.
- Approval by Board of Directors consisting of the seven chiefs of Battlefords Tribal Council Indian Health Services First Nations through a Tribal Council Resolution and a Band Council Resolution by Saulteaux Chief and Council stating support for the research proposal.

#### Considerations in Community Based Research

Community-based research (such as has been done through the DREAM projects) has a community development component which has the potential for long-term benefits within the community that reach beyond the client participants. Research done in this manner has a number of components that are different than clinic based research; some of these are outlined below.

- Introductory Phase This phase includes the need to increase community awareness and building a common understanding of the project and sets the stage for recruitment. Establishing the credibility and benefit of the research is essential.
  - Processes: Staff discussions with individuals, community information sessions, media announcements, posters, pamphlets and presentations to key groups.
- Trust Issues. One of the critical components for success of the research project to build a foundation of trust and credibility in the research project. The trust development includes the acceptance of the outside investigators’ knowledge, skill, motivation and commitment. This must be established before the recruitment begins.
- Recruitment Phase also has a community-based and personal approach, which requires knowledge of community members and dynamics.
  - Processes
    - Identification of prospective clients who fit criteria through multiple methods which may include: client record reviews, screening clinics, client reviews and discussions with community members.
    - Personal invitations to the identified prospective clients.
    - Clients who express interest are visited to discuss the study in more detail. The consent is explained with special emphasis on research processes (including the research and control arms).
    - Organization of community-based clinics for investigators to see clients for the first time. Each of theses clinics require publicity, invitations, and arranging staffing and space.
- Community Perceptions: Unlike clinical research, community-based research can be detrimentally affected by one or two clients who have negative experiences (real or perceived). If clients or other community members express serious concerns about the project to other community members, the project credibility may be damaged. Community-based staff often pick up the concerns and quickly intervene, or take action to ensure investigators are informed and can assist in corrective action.
- Community Events and Weather: affects the time frames and efficiency of work. When community deaths occur, clinics are postponed out of respect for the family for four days during the wake, funeral and feast period. Adverse weather from cold, snow or impassible mud roads may also cause delay of services or postponement of investigator clinics.
- Workload Considerations: Community-based Home and Community Care staff carry a full load for Home and Community Care services in addition to the research project. Sudden increases in workload caused by high needs or very acute care clients will cause delays in the less critical services required by the research project.
- Community Wide Impacts and Changes: Using the community development approach results in advancement of knowledge and in some cases the health practices of community members who have not been directly involved in the research. DREAM 1, 2 and 3, have demonstrated that as result of community wide exposure to the diabetes awareness and education campaigns, some community members not directly involved in DREAM as clients have shown interest in prevention programs and self care practice changes.
